# Navigating Cognitive Maps: Statistical Analysis of 3D Path Data in Minecraft

**DOI:** 10.1017/psy.2025.10069

**Published:** 2026-01-13

**Authors:** Jizhi Zhang, Alessandra Shuster, Allison B. Morehouse, Sara Mednick, Zhaoxia Yu, Weining Shen, Katharine C. Simon

**Affiliations:** University of California Irvine, USA

**Keywords:** Dijkstra’s algorithm, functional data analysis, functional regression, spatial memory, spatial navigation

## Abstract

Understanding spatial navigation and memory formation is critical to exploring how humans learn and adapt in complex environments. To investigate these processes, we conducted an experiment using the Minecraft Memory and Navigation Task, collecting detailed three-dimensional (3D) path data in a virtual open-world setting. Statistically, we developed a novel methodology to convert complex high-dimensional 3D movement data into functional representations, enabling standardized comparisons and analyses across participants and environments. We applied techniques such as functional clustering and regression to identify navigation patterns and their relationships with cognitive map development and memory retention. Our analysis uncovered two significant insights: first, participants who adopted moderately exploratory behaviors during training demonstrated superior retention of object locations; second, inefficient navigation strategies were strongly linked to poorer spatial memory and navigation performance. These findings highlight the effectiveness of our methodology in advancing the study of navigation behaviors and cognitive processes in dynamic 3D environments.

## 1 Introduction

Understanding how humans navigate new environments, learn spatial relationships, and retain this knowledge for efficient retrieval is critical to unraveling human behavior and spatial cognition (Jarlier et al., 2013; Peer et al., 2021). Navigation relies on cognitive maps—internalized mental representations of spatial layouts—that facilitate efficient movement and decision-making in complex environments (O’keefe & Nadel, 1978; Peer et al., 2021). Traditional studies in this domain often employ constrained environments, such as mazes or cities with fixed pathways, which, while useful, are limited in their ability to replicate the variability and complexity of real-world navigation (Ugwitz et al., 2019). The lack of real-world complexity limits our understanding of how individuals adapt their navigation strategies and form flexible cognitive maps to reach their goals in dynamic and unpredictable environments.

Spatial navigation is typically assessed using metrics, such as route accuracy, heading trajectory, path efficiency, map building, or time-to-goal measures (Simon et al., 2022). Additionally, the difference in steps or time between a chosen route and an optimal route is commonly used to gauge knowledge of the environment. These metrics are often analyzed using linear statistical measures, which provide a simplified view of navigation performance (Jarlier et al., 2013; Mohaddesi et al., 2025). However, these approaches fail to capture the dynamic learning, the adaptive shifts in navigation strategies, and the evolving spatial relationships essential for developing a cognitive map over time (Chrastil & Warren, 2015).

To address these limitations, our study employs an open-world, three-dimensional (3D) experimental framework using the Minecraft Memory and Navigation Task (MMN) that offers an ecologically valid setting for exploring how individuals navigate, learn, and retain spatial information (Simon et al., 2022). Unlike prior studies that rely on environments with predefined routes or constrained navigational choices (Jarlier et al., 2013; Pecchioli et al., 2011), our environment replicates real-world conditions, incorporating features, such as open exploration, complex terrain, and dynamic challenges. This approach enables us to examine navigation behavior not only as a static process, but as a dynamic series of behaviors during learning and retention.

Our methodological contribution to the field of spatial cognition lies in our novel statistical approach to quantifying open-field movements of players during the learning and testing of unique object–location associations using the MMN (Simon et al., 2022). In this task, players initially explore an environment to find and learn the locations of unique objects. Their goal is to remember these specific locations, as they are later tested on their ability to recall them. A key innovation in our approach is our application of advanced statistical methods to analyze players’ navigation trajectories during learning and testing. Rather than simply assessing movement directness, we also consider the progression of learning object–location associations and the flexible use of navigation strategies while searching. To effectively process these movement data, we leveraged Dijkstra’s algorithm (Dijkstra, 2022), which has been widely used in real-world navigation and topographical analysis for the use of unmanned vehicles (Dawid & Pokonieczny, 2021; Del Mondo et al., 2013; Martin et al., 2023).

The Dijkstra algorithm models an environment as a weighted graph, where the nodes represent terrain blocks in Minecraft, and the edges represent the movement costs, which are influenced by terrain features. For instance, much like in the real world, vertical mobility in Minecraft is limited: players cannot simply jump up a mountain but must ascend block by block, or a few blocks at a time. Additionally, the algorithm accounts for in-game penalties, such as damage from steep falls, which impose a higher cost on certain descents. This approach accounts for both the physical limitations of engaging in a 3D world, similar to real-world terrain, and the cognitive processes that players experience while navigating the environment (Dawid & Pokonieczny, 2021; Del Mondo et al., 2013; Martin et al., 2023). To quantify a player’s navigation behavior while searching for hidden objects throughout the world, we introduce a new definition called the *cost difference curve*, which represents the cost difference between the actual compared to optimal movement taken by a player between two objects during learning. These segments, as we term them, simplify the movement between two objects during learning into a form suitable for further analyses. Benchmarks, defined as optimal costs for traversing specific object-to-object segments, facilitate comparisons across participants and environments. In this framework, high-dimensional 3D path data are converted to densely observed functional data defined over a unified time interval, allowing for advanced analyses, including functional clustering and regression (Ramsay & Silverman, 2002), providing valuable insights into behavioral navigation strategies and patterns.

Our study explores the development of cognitive maps, drawing on theories that propose that spatial knowledge is encoded as both Euclidean maps and graph-like representations (Peer et al., 2021). These dual representations allow for flexible adaptation to novel challenges, whether through the use of global spatial relationships or localized route connections. By investigating how representations of location–environment relationships emerge in our open-world framework, we aim to provide a deeper understanding of the mechanisms underlying the building of cognitive maps. We assess navigation strategies during learning and immediate testing to determine which search behaviors predict retention. On the one hand, participants could apply a consistent strategy across learning. Alternatively, participants may be inconsistent in their navigation strategies, switching between behaviors that appear to engage allocentric or egocentric cues. Allocentric navigation relies on external references, such as landmarks or cardinal directions, whereas egocentric navigation depends on the individual’s own body-centered frame of reference. Thus, our study addresses how spatial environmental relationships are acquired to support the development of an overarching cognitive map (Jarlier et al., 2013) and also examines the implementation of navigational strategies while adapting to task demands (Krichmar & He, 2023; Mohaddesi et al., 2025).

All analysis scripts, preprocessing pipelines, and simulation code used in this study are openly available at the following repository:

https://github.com/Zhangjizhi2022/minecraft-navigation.

## 2 Methodology

### 2.1 Study design

The experiment utilized the Minecraft Memory and Navigation task (MMN), which is composed of four distinct terrain environments (Simon et al., 2022). Relevant data from this task have been reported in the referenced study. This study was approved by the Institutional Review Board at the University of California Irvine. All participants provided verbal and written consent prior to participation. Participants received either course credits or monetary compensation for their time.

### 2.2 Participants

In total, 182 participants completed the Minecraft Memory and Navigation Task (see Table 1a and 1b for details). All participants were healthy adults with no reported medical, mental health, or sleep disorders. For each participant, in addition to their performance in the game, we also recorded a set of baseline covariates to better understand individual differences and their potential influence task performance. Table 1a summarizes all categorical covariates. In Table 1a, Env1, Env2, Env3, and Env4 refer to the specific environment to which participants were randomly assigned. Video game experience refers to participants’ usual self-reported gaming type. First-person games have an immersive perspective in which players view the world of the game through the eyes of the character. Third-person games, on the other hand, offer an external perspective of the character, providing a broader view of the surroundings. We note that more than half of the participants have experience in both game types. Participants reported neither if they did not have experience with either type of video game. Minecraft skill refers to self-reported skill level of the participants specific to Minecraft. More than half of the participants do not have prior game experience in Minecraft. For variable sex, female participants are more than double the male participants. Six participants identified themselves as transgender or bisexual. Table 1b shows the numerical covariates. Gaming hours record the self-reported weekly playing time, and age refers to the participants’ age in years. These covariates allow for detailed exploration of factors influencing performance and behavior within the experiment.

**Table 1:** Descriptive statistics of participant covariates across virtual environments

Variable	Category	Env1	Env2	Env3	Env4
(a) Distribution of categorical covariates by environment					
Count	-	45 (100%)	53 (100%)	42 (100%)	42 (100%)
Gaming experience level	Expert	13 (28.9%)	11 (20.8%)	8 (19.0%)	12 (28.6%)
	Intermediate	15 (33.3%)	18 (34.0%)	13 (31.0%)	15 (35.7%)
	Novice	17 (37.8%)	24 (45.3%)	21 (50.0%)	15 (35.7%)
Sex	Female	27 (60.0%)	38 (71.7%)	30 (71.4%)	28 (66.7%)
	Male	17 (37.8%)	14 (26.4%)	9 (21.4%)	13 (31.0%)
	Other	1 (2.2%)	1 (1.9%)	3 (7.2%)	1 (2.4%)
Minecraft skill	Expert	8 (17.8%)	4 (7.5%)	5 (11.9%)	6 (14.3%)
	Intermediate	13 (28.9%)	14 (26.4%)	8 (19.0%)	18 (42.9%)
	Novice	24 (53.3%)	35 (66.0%)	29 (69.0%)	18 (42.9%)
Video game experience	Both	21 (46.7%)	25 (47.2%)	18 (42.9%)	29 (69.0%)
	First-person	9 (20.0%)	12 (22.6%)	10 (23.8%)	4 (9.5%)
	Neither	8 (17.8%)	9 (17.0%)	7 (16.7%)	6 (14.3%)
	Third-person	7 (15.6%)	7 (13.2%)	7 (16.7%)	3 (7.1%)

(b) Distribution of continuous covariates (mean and SD) by environment				
Variable	Env1	Env2	Env3	Env4
Age (years)	21.09 (1.83)	21.49 (4.73)	21.05 (3.36)	20.97 (1.74)
Gaming hours	1.51 (1.78)	1.53 (1.84)	1.29 (1.61)	1.54 (2.02)

### 2.3 Minecraft memory and navigation task

**Figure 1:**
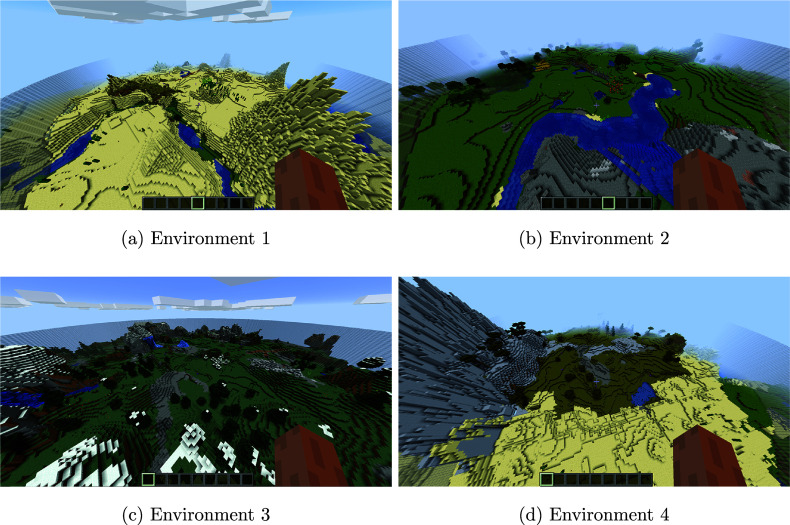
Screenshots of four game environments.

Each of the four environments was a fully immersive 3D space, providing participants with a realistic and interactive experience. These 3D environments were designed to ensure consistent complexity and layout while maintaining distinct spatial configurations to minimize potential carryover effects between environments. Each environment contained 12 distinct objects, along with obstacles and impassable tiles, such as fences, rivers, cacti, and trees, which added an additional layer of challenge and realism. Participants were randomly assigned to one of the environments to ensure balanced conditions throughout the study. Participants were only allowed to walk or run during the task. Screenshots for each of the environments are shown in Figure 1.

The MMN task had two primary aims. The first aim was to familiarize participants with the virtual environment and evaluate their ability to search through an environment and encode the spatial relationships between objects, landmarks, and boundaries. This phase assessed participants’ capacity to form a cognitive map through free exploration. The second aim was to test and measure participants’ spatial memory and navigation strategies by requiring them to recall and accurately place objects within the environment. Each participant completed two training sessions and one test session. The methodology of the MMN task is briefly described below; for more detailed information, please refer to Simon et al. (2022).

*Training.* During the training session, the participants began at a central starting point within their assigned environment and were instructed to explore freely. Their task was to locate all 12 objects, hidden within chests in the ground and marked by a single pink block above, and memorize their locations. The object locations were not provided to the participants during training or test. The training phase ended when participants either found all 12 objects or the allotted time of 10 minutes per training expired. Participants could have up to 20 minutes of free exploration. Immediately after completing the first training session, participants proceeded to the second training session and repeated the same procedure.

*Test.* Following the two training sessions, the participants were immediately tested on their knowledge of the location of the 12 objects. The order in which the objects were presented during the test was randomized. Furthermore, to increase the task’s difficulty, there were four potential starting points, on the outer edge of the environment in the test phase, which differed from the start location in the training phase. For each test trial, the participant was teleported randomly to one of the four test start locations and instructed to place the testing object at the location they remembered from the training. After placing the object, the participant was again teleported to a new location and the next object to be tested was provided. Participants were given a maximum of 3 minutes to place each object during a test trial.

### 2.4 Spatial memory and navigation task data

During both training and test sessions, participants’ in-game 3D coordinates were recorded each second to track their movements. In addition, a log file was maintained to capture specific timestamps for key events. In the training sessions, the timing began as soon as the participants entered the environment. Each time a participant found an object, the exact time was recorded, creating a detailed log of their progress. For the test session, for each of the 12 objects, we recorded the exact time participants entered the environment after being presented with the specific object, the time, and location of their object placement.

## 3 Statistical analyses and results

### 3.1 Navigation in 3D environments: Past work and challenges

The 3D environment in Minecraft provides significantly richer information compared to traditional two-dimensional (2D) spaces due to its distinct vertical dimension. Unlike the horizontal plane, vertical movement is heavily constrained, as participants are generally bound to the ground of the Minecraft world. This limitation means that the vertical dimension cannot simply be treated as an extension of the other two dimensions, as free movement upward or downward is not possible. The rules of the Minecraft game further emphasize these restrictions, e.g., players cannot ascend cliffs or vertical surfaces higher than three blocks without the aid of tools or structures, which introduces a layer of strategic planning in navigation. Similarly, falling from significant heights results in health penalties, making the vertical dimension not only a physical but also a consequential element of game play.

These constraints also create a distinct cognitive experience for the players. Just like in the real world, individuals naturally favor flat or gently sloping terrain over steep or rugged landscapes, reflecting an intuitive understanding of effort and risk. Moreover, unique or striking topographical features tend to leave lasting impressions, enhancing memory and shaping navigation patterns within the environment. The interplay between verticality, terrain, and cognition highlights how Minecraft’s 3D environment offers a more complex and immersive experience than its 2D counterpart.

**Figure 2:**
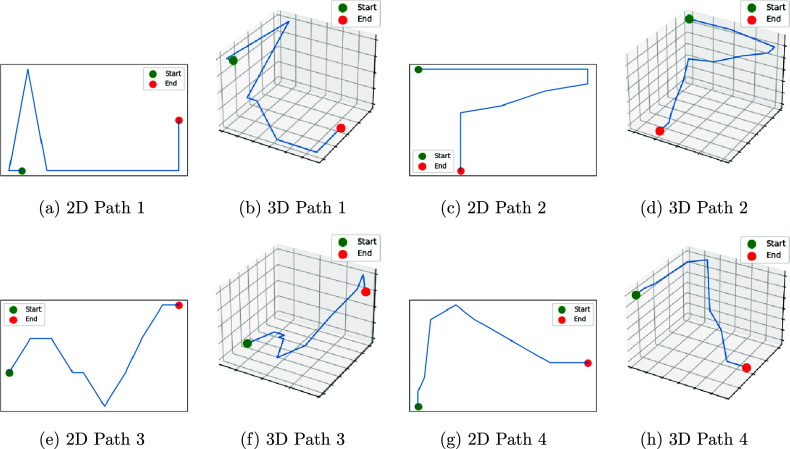
An example of comparisons between 2D and 3D paths.

We use Figure 2 to demonstrate the advantage of considering 3D paths over their 2D versions. For each pair of pictures, we note that 3D paths provide more information and naturally help with better understanding study participant’s in-game behavior. For example, in 2D pictures, participants often take seemingly indirect or circuitous routes despite the availability of straightforward paths. However, by checking the 3D pictures, it becomes evident that the participants are actually attempting to avoid steep inclines or declines by taking alternative routes, thereby minimizing the slope and reducing the effort required for movement.

We highlight a few statistical challenges when analyzing 3D path data in our study. First, 3D paths are inherently high-dimensional. For example, a participant who spends 10 minutes in one training session would result in 600 longitudinal measurements of 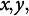 and *z* coordinates. This has not even counted for additional variables, such as time stamps and speed information. Moreover, the movement data are highly auto-correlated in both space and time, which makes common dimension reduction techniques, such as PCA and t-SNE (Nathan et al., 2008; Troje, 2002) not directly applicable. Third, one needs to take into account the game environment and rules when analyzing the data. For example, the constraints of the third dimension, such as limited vertical mobility and penalties for steep slopes or falls, differ significantly from the first two dimensions. For this reason, several popular approaches in spatial statistics, such as state-space models (Howard et al., 2008), Gaussian process-based approaches (Nardi & Stachniss, 2019), and spatial–temporal approaches (Lu et al., 2022) are not directly applicable. Lastly, path lengths depend on several factors, such as task difficulty (e.g., paths between different pairs of objects may have different difficulty levels) and participant heterogeneity (e.g., different navigation strategies, and game skill levels). Classical curve alignment methods, such as Dynamic Time Warping (Müller, 2007) are not directly applicable due to these factors.

### 3.2 Defining optimal paths

Navigating and analyzing 3D data pose unique challenges that differ significantly from those in 2D environments. In 3D spaces, relying solely on 2D data oversimplifies the environment, failing to capture critical aspects of player behavior. Moreover, applying standard 3D Euclidean distances is insufficient, as constraints on the third dimension—such as limited vertical mobility and penalties for steep slopes or falls—often diverge from those of the horizontal dimensions.

To address these challenges, it is crucial to use a pathfinding algorithm that can flexibly model cost under environmental constraints. A natural choice is *Dijkstra’s algorithm* (Dijkstra, 2022), a classic method in graph theory known for computing the shortest path in a weighted network. More details of Dijkstra’s algorithm are provided in Appendix A. By explicitly modeling movement costs and constraints, such as elevation or hazardous terrain, Dijkstra’s algorithm can be readily adapted to Minecraft’s 3D environment. This flexibility makes it invaluable for solving navigation problems where standard Euclidean metrics are insufficient. Its applications span GIS, robotics, and spatial behavior modeling (Saab & VanPutte, 1999), providing both accuracy and computational efficiency in complex, constrained spaces.

Our work builds on prior research that uses graph-based models to study trajectory data (Spaccapietra et al., 2008). These models, which represent movement paths as graphs with nodes for locations and edges for movements, have been successfully applied in domains like motorized transportation and unmanned vehicles (Del Mondo et al., 2013). One of the key advantages is the flexibility to incorporate additional spatio-temporal constraints, as well as the ability to model and analyze spatio-temporal evolutions (Elayam et al., 2022; Martin et al., 2023). By leveraging these graph-based techniques, we aim to address the complexities of 3D navigation while ensuring accurate modeling of player movement in dynamic environments.

For our Minecraft data, we use the algorithm to evaluate all feasible (walkable) paths and pinpoint the shortest route between them. We begin by introducing the concept of a block. In Minecraft, a block is a cube that measures one unit on each side. It is the fundamental unit of the game world as it serves as the basic building and interaction element. Blocks are aligned to a grid, meaning that their positions are fixed to integer coordinates. Consequently, the entire game world is structured as a 3D grid of blocks.

To describe the application of Dijkstra’s algorithm in our study, we start with a 3D environment terrain map 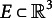 . A Minecraft block 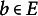 is represented as 
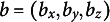
 , where 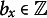 and 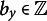 are integers corresponding to the block’s 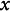 - and 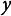 -coordinates, respectively, and 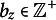 is a positive integer uniquely determined by 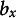 and 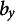 . Together, these coordinates define the block’s location in the 3D environment. In our model, blocks serve as nodes in a graph, with edges connecting adjacent blocks based on specific criteria that reflect the mechanics of Minecraft’s game. The edge from block 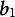 to block 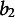 is considered walkable if the following conditions are satisfied:

1. *Both blocks must be valid and reachable:* 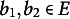 . This ensures that both blocks belong to the terrain map and are not obstructed by elements, such as water, trees, or fences, which would render them inaccessible within the game environment.
2. *Blocks must be adjacent in a 2D sense:*


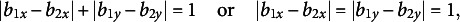

  i.e., adjacency is defined as direct neighbors (in *x* or *y* direction) or diagonal adjacency.
3. *Vertical height changes must be within a permissible range:*


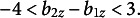

  This constraint indicates that, in the Minecraft environment, players cannot climb cliffs higher than two blocks or descend falls exceeding three blocks in a single step.

These rules ensure that the graph accurately represents the navigational constraints and possibilities within the 3D Minecraft environment, forming the basis for applying Dijkstra’s algorithm to find the optimal paths. With these rules in place, we can now define the distance or edge weight between two walkable nodes. The distance is defined as follows: (1)

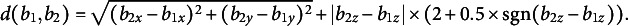



The first term in (1) is the standard 2D Euclidean distance between the two blocks, while the second term corresponds to the additional cost associated with vertical movement. Here, sgn 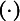 is the sign function. Definition (1) ensures that the 2D distance remains consistent with the standard Euclidean distance in two dimensions, while the cost increases with the height difference to reflect the greater effort required to navigate steeper vertical transitions. Additionally, moving upward incurs a higher cost than moving downward, aligning with the physical and cognitive challenges associated with climbing.

For a given environment *E*, we defined a weighted graph based on the terrain and its properties discussed above. Using this graph, for any pair of blocks 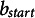 and 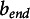 in *E*, we can apply Dijkstra’s algorithm to identify the shortest path between them. The distance or cost of this shortest path, as computed by Dijkstra’s algorithm, is referred to as Dijkstra’s distance, denoted as 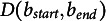 . This distance captures the minimal cost required to traverse from 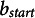 to 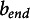 .

### 3.3 Cost difference during object–location learning

**Figure 3:**
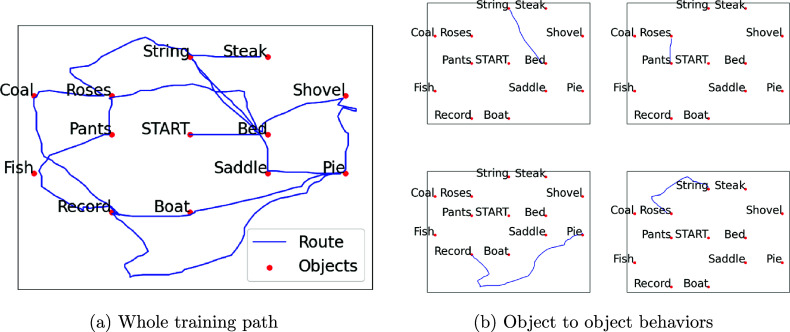
Example of paths from participant no. 2025 during training session 1.

Although Dijkstra’s algorithm provides a connected terrain map, analyzing individual behavior reveals additional complexities that require further interpretation. For example, Figure 3 illustrates an inconsistency in a player’s training session while learning object locations. In some object-to-object movement, such as moving from the Bed object to the String object or moving from the Pants object to the Roses object, the player appears to walk along a clear, efficient path toward a distinct destination. In contrast, in other movements, such as from the Pie object to the Record object or from the Roses object to the String object, the player exhibits wandering behavior, deviating from a straightforward path and covering significantly more distance than necessary. This variability suggests the need to distinguish and classify different search behavior patterns within a single player’s movements during learning.

We now segment the training path—the players complete movements during learning—into distinct segments to better analyze individual behavior. Each segment is defined as starting at one unique object and ending at the next, allowing for a structured breakdown of the path. Once segmented, each segment can be analyzed independently for key metrics, such as cost, distance, and elevation changes. These metrics provide a basis for further analysis, including clustering and association studies.

For a segment 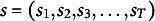 of participant *i*, with 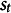 representing the 3D coordinate at time 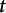 starting from a certain object, and 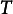 representing the total time spent moving from one object to another, we define the *actual cost* of the segment 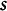 from time 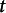 as 

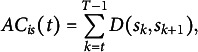



where 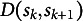 represents the cost of moving from coordinate 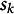 to 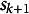 . We also define the *optimal cost* of the segment 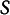 from time 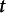 as 

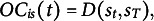



which represents the minimum possible cost of moving directly from 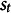 to 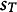 .

For any segment 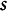 of participant *i* and time 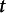 , it can be seen that 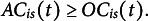 This is because 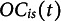 represents the smallest possible cost of traveling from 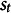 to 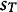 , as it corresponds to the distance computed using Dijkstra’s algorithm; while 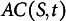 refers to the cost of a specific path taken to move between the same points, which may involve additional deviations or detours. As a result, it follows naturally that 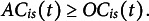


Now, we define the *cost difference* of a segment *s* of participant *i* at time 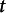 as 

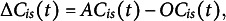



where 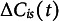 represents the difference between the actual cost and the optimal cost of moving from 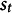 to 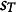 . Here, 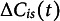 can be viewed as a non-negative function of time observed at 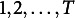 . This definition helps isolate segment-specific behavioral patterns, decoupling them from the original terrain and object location data. This decoupling simplifies the data structure while standardizing comparisons across segments, making it easier to identify patterns and trends independent of the original environment.

To ensure consistency across all segments, we normalize the cost difference curves. This step involves aligning all curves to the same length, as the duration of movement between objects can vary substantially across segments. More specifically, let 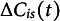 be the cost difference curve of a segment 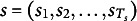 with total duration 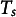 for participant *i*. We normalize this curve by interpolating it to a fixed time domain 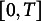 , where *T* is a unified time scale set as 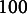 in our study. We denote the normalized curve by 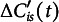 , which is a function of rescaled time 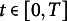 . It is important to note that normalizing the curves does not result in significant information loss. Although the original lengths of the curves may reflect different behavioral patterns—shorter curves often represent more direct paths and longer curves indicate more circuitous routes—the process of normalization retains the essential shape and variation of the cost difference. The underlying patterns of behavior, such as whether a participant followed an efficient or wandering path, remain embedded in the structure of the normalized curves. This ensures that critical behavioral information is preserved for subsequent analysis.

### 3.4 Clustering cost difference curves

We employ a functional clustering approach to analyze the normalized cost difference curves. This methodology follows a widely used framework in functional data analysis that combines basis function representation with centroid-type clustering methods. In particular, prior work has integrated B-spline or wavelet bases with *K*-means or fuzzy clustering algorithms to cluster functional trajectories (Abraham & Cornillon, 2003; Giacofci et al., 2013; Jacques & Preda, 2014). Our approach adopts a similar strategy by using cubic B-spline basis functions to extract coefficient vectors from each normalized curve, followed by *K*-means clustering applied to the estimated coefficients. This setup enables the identification of prototypical navigation patterns across segments while maintaining computational simplicity. To capture common patterns, we combined all cost difference curves, regardless of environment or training phase, into a single dataset prior to clustering. We first smooth the curves using spline analysis to reduce noise while preserving key structural features. Splines are particularly suited to this task because we are primarily interested in the turning points within the curves, which represent critical behavioral patterns. We use cubic spline interpolation (Ramsay & Dalzell, 1991) for convenience (we have tried higher-order splines but the results are largely similar so we decide to keep the spline order at 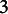 ). The interval 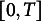 is divided into sub-intervals with equally spaced knots. We denote the corresponding spline basis functions by 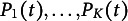 . A total of 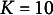 basis functions were used. We have conducted a sensitivity analysis on this choice in Appendix B and the results suggest that the clustering results are not sensitive to this choice. For each normalized cost difference curve 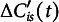 , we can approximate it by a linear combination of spline basis functions: (2)

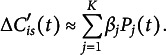



The spline-based approach highlights transitions and curvature, aligning with our goal of understanding individual behavioral variability. We estimate the coefficients 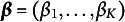 using least squares fitting for each of the normalized cost difference curves and then perform *K*-means clustering (Jacques & Preda, 2014) to the estimated spline coefficients. It is important to note that each study participant has about 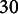 curves corresponding to 30 path segments from two training sessions combined. Clustering is performed at the curve level instead of the participant level because we are mainly interested in understanding the heterogeneity among the path segments. In the next section, we will conduct a participant-level analysis.

**Figure 4:**
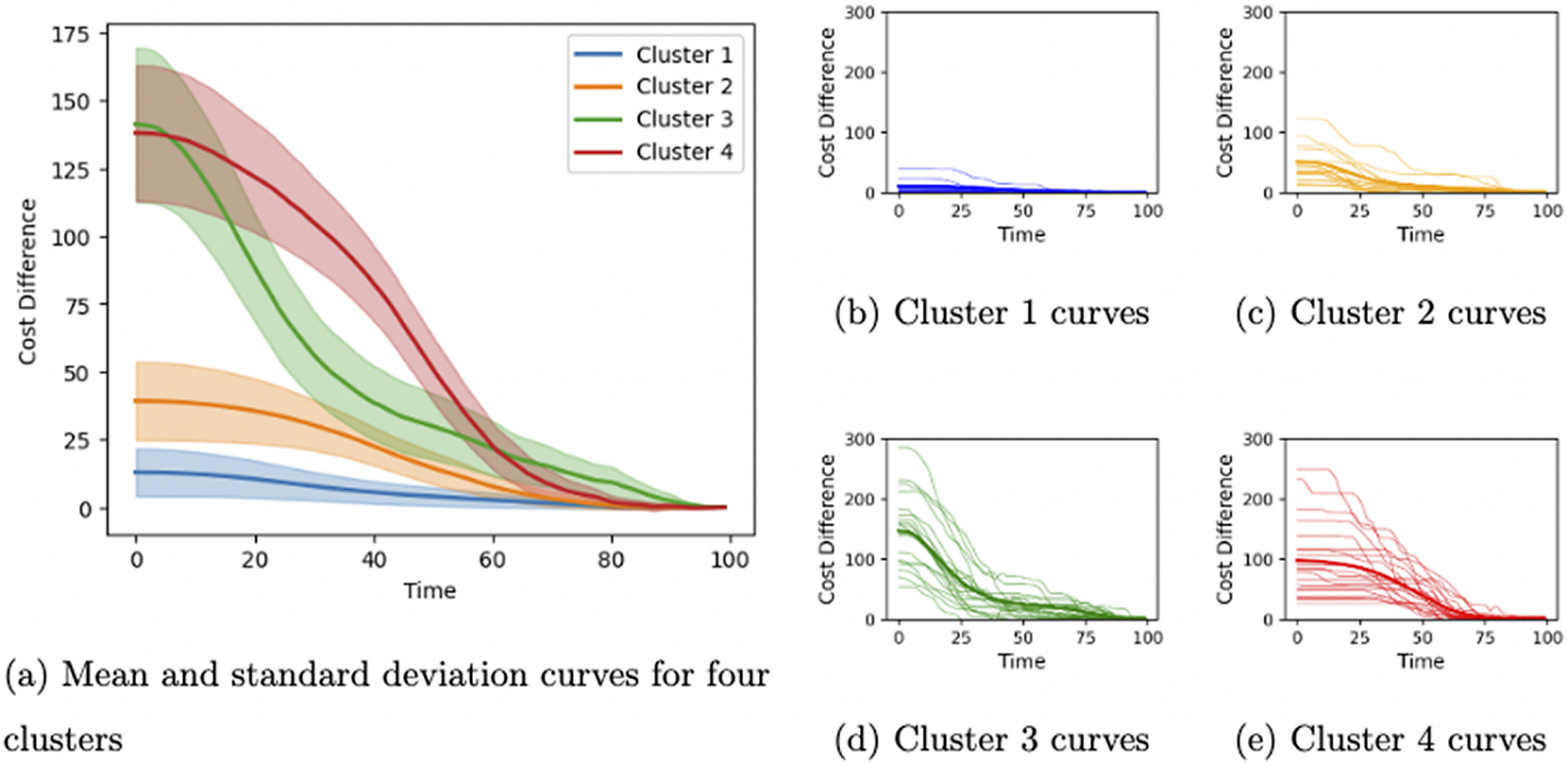
Functional clustering analysis results.

Through our clustering process, we have identified four distinct clusters representing different types of trajectories based on their cost difference curves. The number of clusters is determined by the Silhouette score (an elbow plot is provided in Appendix E). Each cluster captures unique behavioral patterns, shedding light on the variability in navigation strategies among participants. Figure 4a shows the mean and standard deviation curves for each cluster. There are a total of 5,572 curves, and 4,499 curves (80.74%) in Cluster 1, 826 curves (14.82%) in Cluster 2, 121 curves (2.17%) in Cluster 3, and 126 curves (2.26%) in Cluster 4. To check the stability of our clustering result, we repeat the same clustering analysis 1,000 times with different random seeds and calculate the adjusted Rand index (ARI) between these cluster results and the current one. The average ARI is 0.9973, with a standard deviation of 0.0059. This confirms the stability of our cluster results.

**Figure 5:**
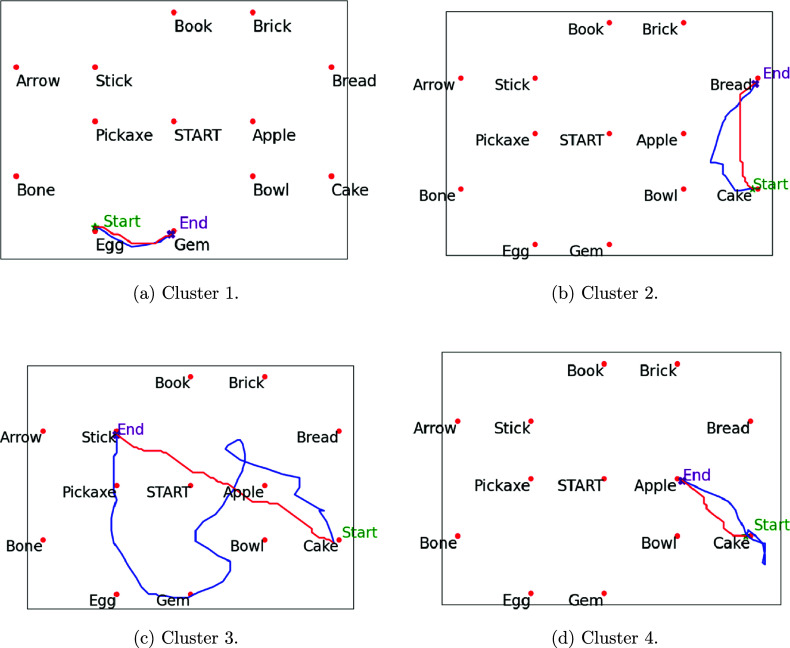
Comparison of sample curves to their optimal paths for each of the four clusters. *Note*: Each subfigure represents a cluster and it contains four different environments.

Cluster 1 (Figures 4b and 5a) represents the best-performing trajectories, characterized by minimal cost differences. This cluster dominates, encompassing over 80% of the cost difference curves. These trajectories reflect the most efficient behavior, where participants move directly from one object to the next with a clear target in mind. The paths in this cluster suggest a high level of decisiveness and effective navigation, indicating that for most segments, participants follow an optimal or near-optimal route.

Cluster 2 (Figures 4c and 5b) captures the second-best trajectories, which exhibit moderate improvements in efficiency over time. Although these paths are less direct than those in Cluster 1, they show evidence of participants adjusting their behavior as they navigate. The moderate cost differences suggest that participants may initially deviate from the optimal path but eventually correct their course. This cluster likely represents learning or exploratory behavior, where participants refine their routes as they gain more information about the environment.

Clusters 3 and 4, on the other hand, represent poor-performing trajectories with significant inefficiencies. These trajectories deviate substantially from the optimal path, indicating struggles in navigation. Cluster 4 (Figures 4e and 5d), in particular, highlights a pattern in which participants initially struggle to find the correct path, but quickly adjust and align with the optimal trajectory. This cluster suggests a reactive behavior, where participants make abrupt course corrections after recognizing inefficiencies in their initial navigation choices. The cost differences drop sharply before the middle of the segment, reflecting this rapid adaptation.

In contrast, Cluster 3 (Figures 4d and 5c) displays prolonged inefficiency. Participants in this cluster take significantly longer time to identify the correct path, resulting in consistently higher cost differences for the most part of the segment. This cluster may reflect hesitation, confusion, or repeated errors in decision-making. The trajectories in Cluster 3 highlight the challenges some participants face in navigating the environment effectively, providing an interesting contrast to the adaptive behavior seen in Cluster 4.

To explore the impact of training on cluster distributions, we analyzed the proportions of cost difference curves in each cluster for two training datasets. From Table 2, we observe a notable difference between Training 1 and Training 2, particularly in Cluster 1 and Cluster 2. Cluster 1, which represents the best-performing trajectories, shows a higher proportion in Training 2 (82.9%) compared to Training 1 (78.6%). In contrast, Cluster 2, which captures moderately efficient trajectories, decreases from 16.9% in Training 1 to 12.7% in Training 2. This suggests that some trajectories from Cluster 2 in Training 1 may have switched to Cluster 1 in Training 2, indicating improved navigation efficiency. Clusters 3 and 4, representing less efficient trajectories, exhibit minimal changes across the two trainings. Cluster 4 shows very low proportions in both datasets, with a slight decrease in Training 2 (2.3% vs. 2.0%), while Cluster 3 remains nearly constant.

**Table 2:** Cluster distribution for two training sessions

Training	Cluster 1	Cluster 2	Cluster 3	Cluster 4
1	78.6	16.9	2.2	2.3
2	82.9	12.7	2.3	2.0

These findings support the hypothesis that Training 2, conducted immediately after Training 1, benefits from participants’ increased familiarity with the environment. The higher proportion of efficient trajectories in Cluster 1 and the reduction in moderately efficient trajectories in Cluster 2 demonstrate improved performance and adaptation over time. This improvement highlights the impact of prior exposure on the game and demonstrates a learning benefit between consecutive training sessions.

The data in Table 3 also highlight the impact of different environments on participants’ behavior. Notably, the fourth environment shows a marked difference compared to the first three environments. In Environment 4, the proportion of trajectories in Cluster 1 drops to 76.8%, compared to over 81% in the other environments. This indicates that participants were less likely to adopt the most efficient behaviors in this environment. The results imply that the distinct characteristics of Environment 4 may have posed additional challenges or encouraged exploration, leading to a slight shift in behavior patterns and small decrease in efficient movement.

**Table 3:** Cluster distribution for four game environments

Environment	Cluster 1	Cluster 2	Cluster 3	Cluster 4
1	81.7	14.2	2.0	2.2
2	82.6	13.3	2.2	1.8
3	81.6	13.9	2.3	2.2
4	76.8	18.0	2.2	3.0

Table 4 provides insight into the difference in navigation patterns between objects first found during training compared to at the end. The “first segment” refers to the segment where a participant begins the training session, starting from the initial point and moving toward the first object. In contrast, the “last segment” represents the transition from the penultimate object to the final object in the session. After completing the “last segment,” the training session concludes. These segments are particularly significant as they mark the temporal boundaries of the training process and may exhibit distinct behavioral patterns compared to the intermediate segments.

**Table 4:** Cluster distributions for all segments (all), path segments to the first subject (first), and segments to the last subject (final)

Segments	Cluster 1	Cluster 2	Cluster 3	Cluster 4
All	80.8	14.8	2.2	2.2
First	90.6	8.2	0.7	0.5
Last	80.1	11.7	4.0	4.2

The first segment, where participants are just beginning to navigate the environment, shows a significantly higher proportion of trajectories in Cluster 1 (90.6%). This suggests that participants tend to make direct and efficient choices when selecting their initial target, likely opting for the nearest object. The high efficiency of these paths reflects the relatively straightforward decision-making process early in the task, as participants have not yet been influenced by exploration or challenges introduced by subsequent targets. In contrast, the last segment, where participants are finding the final remaining object, which is often more challenging to locate. This difficulty is reflected in the higher proportions of Cluster 3 (4.0%) and Cluster 4 (4.2%) trajectories in the last segment. These clusters represent less efficient navigation patterns, with greater wandering or prolonged decision-making likely due to the increased difficulty in identifying and reaching the last object. These results highlight distinct behavioral patterns associated with the first and last segments. The first segment is characterized by a preference for efficiency and directness, while the last segment reflects greater difficulty and variability in navigation strategies.

In summary, the functional clustering of standardized cost difference curves revealed distinct behavioral patterns in navigation strategies. The results showed that the majority of trajectories (Cluster 1) were characterized by highly efficient and direct paths, suggesting that participants predominantly displayed optimal or near-optimal navigation. Cluster 2 highlighted moderate improvements and adaptive learning, indicating participants’ ability to refine their routes with increased familiarity. Clusters 3 and 4, on the other hand, captured less efficient behaviors, with Cluster 4 reflecting reactive adjustments and Cluster 3 exhibiting prolonged inefficiencies due to hesitation or confusion.

Further analysis demonstrated the influence of training, environmental complexity, and segment characteristics on cluster distributions. Notably, Training 2 resulted in a higher proportion of efficient trajectories, underscoring the role of learning and adapting navigation strategies. The variations across environments revealed that specific environmental features posed some additional challenges, encouraging exploratory behavior. Lastly, the first and last segment effects highlighted distinct temporal-based decision-making strategies during individual training sessions: initial segments showed a tendency for efficiency, while last segments reflected increased difficulty and variability. Together, these findings provide a comprehensive view of navigation dynamics, shedding light on how participants balance efficiency, adaptation, and exploration under varying conditions.

We also repeat our clustering analysis of cost difference curves using functional principal component analysis scores. The resulting clusters are provided in Appendix C and they are very similar to the ones obtained by the B-spline basis. These findings suggest that our analysis is not sensitive to the choice of basis functions.

### 3.5 Functional regression analysis

**Figure 6:**
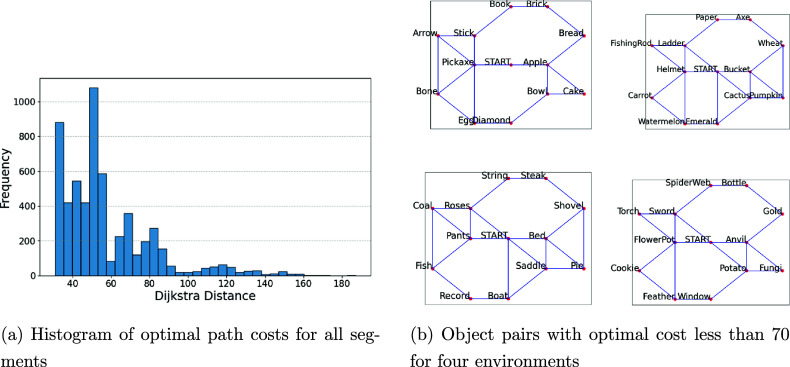
Analysis of optimal path costs across different environments.

To address the open question of how training behaviors influence performance during testing, we aim to extend our analysis from individual segments to a participant-level perspective, which requires aggregating multiple cost difference curves for each participant. This is not a trivial task because of the high level of heterogeneity in these curves. Our strategy is to first perform a classification step on the associated segments by accounting for their intrinsic difficulty levels, i.e., curves from “easy” segments should not be directly combined with curves from “difficult” segments. For each segment, we calculate the cost associated with the optimal path as the Dijkstra distance between the start and end points. In Figure 6a, we show the histogram of optimal path costs for all segments in the training. We decide to use a cutoff of 70 to distinguish between low-cost and high-cost segments. In other words, segments with an optimal cost below 70 are typical paths we expect participants to pick, while segments with an optimal cost above 70 represent inefficient or challenging paths (e.g., two objects are far apart from each other or this segment belongs to a challenging terrain). The cutoff value of 70 is chosen for three reasons: (1) the histogram suggests that most of the segments have an optimal path cost below 70; (2) in all four environments, it is possible to connect between all object pairs with optimal costs less than 70 across. As shown in Figure 6b, these low-cost segments are sufficient to cover the entire map in a relatively direct and efficient manner. (3) 53 of 182 (29.12%) participants pick only path segments with an optimal cost below 70.

**Figure 7:**
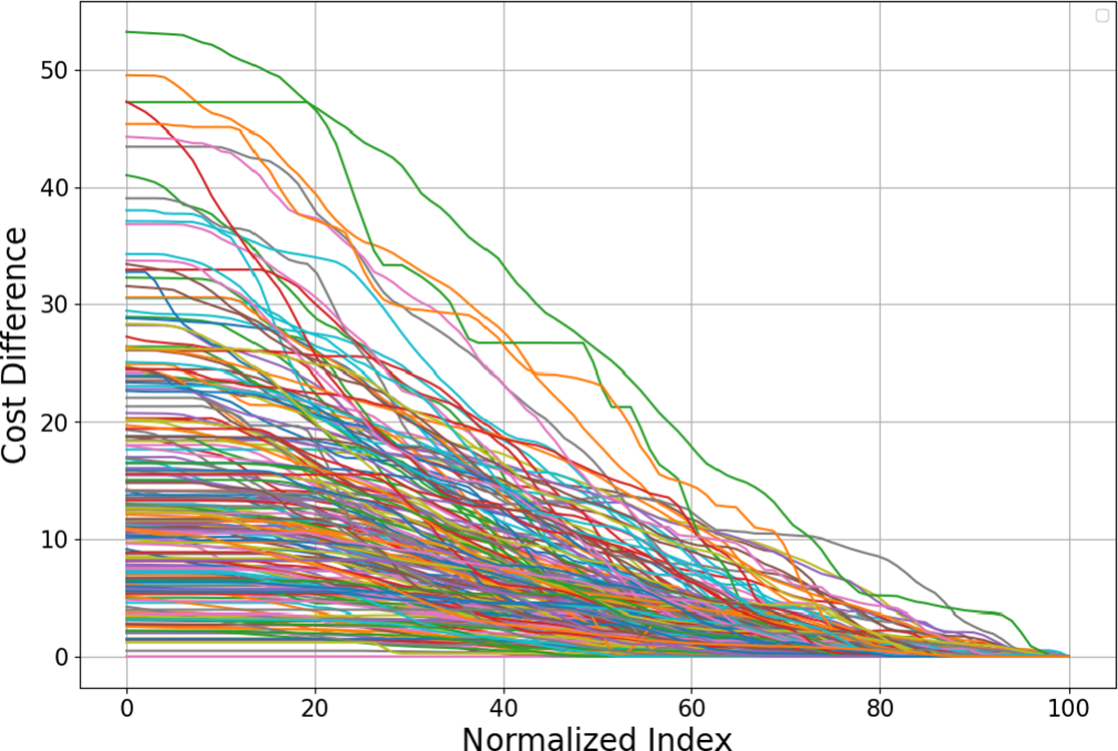
Average cost difference curves for low-cost segments from 182 study participants.

For each participant, we calculate the average curve of the normalized cost difference curves associated with low-cost segments (optimal costs below 70) (see Figure 7 for the average curves of all participants). We also count the number of high-cost segments (optimal cost 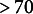 ) for each participant and call it “Removed Curve Count.” This metric provides a complementary perspective, capturing how often participants engaged in inefficient navigation behaviors. By separating the analysis of low-cost and high-cost segments, we aim to better understand the factors contributing to these deviations and their potential impact on overall performance. More discussions about this choice are provided in Appendix D.

To evaluate the test performance of the participants, we define three outcome variables: 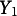 , 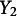 , and 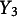 , each capturing a different aspect of navigation and placement behavior. The outcome 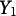 is the total number of objects correctly located during the test. Here, an object is considered successfully located if its placement is within 10-block distance to the object’s true location. This variable measures overall memory, where larger values indicate better performance. The second variable, 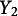 is the average time taken to correctly locate the objects, calculated only for the objects that were successfully placed. This variable reflects efficiency, with smaller values indicating quicker navigation. Finally, 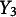 is the average distance from the correct location, measured using the Dijkstra distance, for objects that were correctly placed. This variable represents precision, where smaller values correspond to more accurate placements. By analyzing these target variables, we explore how training behaviors influence test performance and gain insights into the relationship between navigation strategies and outcomes during the test phase.

To model the relationship between training behaviors and test performance, we employ a scalar-on-functional regression (SoFR) model (Crainiceanu et al., 2024; Goldsmith et al., 2011). This approach integrates both scalar predictors, such as participant-specific features, and functional predictors, such as the average cost difference curves. The model can be written as follows: (3)

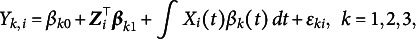

where 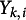 represents the *k*-th test performance outcome for participant 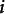 , 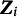 is the set of scalar covariates, including age, sex, video game experience, Minecraft skill, weekly gaming hours, removed curve count, cluster proportions, and average segment time. Cluster proportion is the proportion of clusters 2–4 in each participant’s total segments. The average segment time is the average time a participant spends on all segments during training. The term 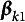 is the vector of associated coefficients. The term 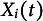 represents the average curve of the normalized cost difference curves associated with low-cost segments for participant *i*, providing dynamic information about a participant’s navigation behavior over time. The functional coefficient 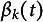 describes how the influence of 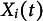 changes over time, enabling us to capture the detailed relationship between the shape of the cost difference curve and the test performance. The error terms 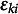 ’s are assumed to be independently and identically distributed (i.i.d.) following a normal distribution 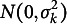 . The model (3) essentially fits separate regressions to each of the 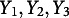 . It is possible to extend this model by considering a joint modeling approach to account for the dependence in the outcomes. We do not pursue this direction here because the correlations among them are small, e.g., Corr 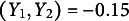 , Corr 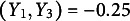 , and Corr 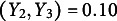 .

We fit functional regressions using the fRegress function from the fda R package. The functional covariate is represented using a B-spline basis with ten basis functions, and the functional coefficients are estimated with a roughness penalty on the second derivative. The penalization is implemented via the fdPar function, using Lfdobj = int2Lfd(2) to penalize curvature and a smoothing parameter of lambda = 0.1. The number of basis functions was set to 10 to allow sufficient flexibility while maintaining smoothness through penalization.

For 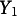 , the total number of objects correctly located, the estimated coefficients for scalar covariates are summarized in Table 5. The model explains a moderate proportion of the variance ( 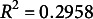 ). The results suggest that sex may influence performance in locating objects. Specifically, males tended to identify more objects compared to females and individuals in the “Other” sex category (estimate = 0.9536, *p* = 0.0569). Additionally, Minecraft skill level significantly affected performance 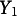 . Participants in the Intermediate skill group tended to locate fewer objects than those in the Expert group (estimate = 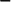 1.2844, *p* = 0.0678), while participants in the Novice group performed significantly worse (estimate = 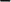 1.7253, *p* = 0.0263). These findings align with intuitive expectations that higher Minecraft skill levels likely correspond to better familiarity with the game mechanics and require lower cognitive demands of video game controls while participating in the task than would be required for a novice player. The proportions of specific clusters also show a notable impact on 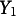 . A higher proportion of segments in Cluster 3 (estimate = 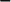 11.6734, *p* = 0.0969) and Cluster 4 (estimate = 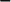 12.3578, *p* = 0.0716), which correspond to less efficient navigation during training, were associated with poorer performance in locating objects during testing. This suggests that these clusters during training capture patterns associated with suboptimal test performance and are indicative of underlying learning factors that negatively affected recall performance.

**Table 5:** Summary of scalar regression coefficients for 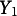 , 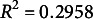

Variables	Covariate	Estimate	Std. Error	*t* value	Pr( 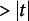 )
Intercept	Intercept	9.0860	1.5210	5.9740	1.4e-08
Sex	Female (Baseline)	–	–	–	–
	Male	0.9536	0.4974	1.9170	0.0569
	Other	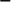 0.6343	1.1292	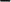 0.5620	0.5751
Video game experience	Both (Baseline)	–	–	–	–
	Third-person	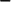 0.5268	0.6528	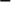 0.8070	0.4208
	First-person	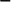 1.1099	0.5846	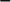 1.8980	0.0594
	Neither	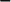 1.8533	0.6771	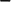 2.7370	0.0069
Minecraft skill	Expert (Baseline)	–	–	–	–
	Intermediate	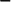 1.2844	0.6986	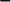 1.8380	0.0678
	Novice	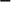 1.7253	0.7695	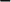 2.2420	0.0263
Continuous variables	Gaming hours	0.0625	0.1255	0.4980	0.6190
	Removed curves count	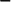 0.1367	0.1656	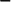 0.8260	0.4101
	Age	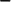 0.0041	0.0616	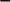 0.0660	0.9473
Environment	1 (Baseline)	–	–	–	–
	2	0.6644	0.5446	1.2200	0.2242
	3	0.3882	0.5806	0.6690	0.5046
	4	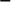 0.9301	0.6017	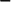 1.5460	0.1241
Cluster proportions	Cluster 2	2.5143	2.5775	0.9750	0.3308
	Cluster 3	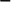 11.6734	6.9921	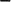 1.6690	0.0969
	Cluster 4	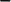 12.3578	6.8138	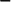 1.8140	0.0716
Time	Time	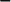 0.0132	0.0332	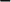 0.3990	0.6907

The influence of video game experience on 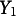 , however, is somewhat unexpected. Compared to participants who played both game types or only third-person games, those who exclusively played first-person games performed worse (estimate = 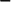 1.1099, *p* = 0.0594). Unsurprisingly, the “Neither” group performed the worst overall (estimate = 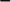 1.8533, *p* = 0.0069).

The scalar estimated coefficients for the functional regression analysis on 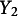 , the average time taken to correctly locate objects, are summarized in Table 6. The model explains a moderate proportion of the variance ( 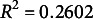 ). However, most covariates were not statistically significant, which may be attributed to the specific definition of 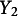 —the average time calculated to successfully locate objects. For individuals who located only a few objects at test, their performance might be influenced by chance rather than by an intentional or efficient navigation strategy. In such cases, their successful placements may result from random or lucky guesses rather than a clear memory or deliberate navigation strategy, thereby diminishing the predictive power of some covariates on 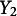 . The Removed Curve Count, however, does exhibit a notable relationship with 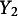 . This variable captures the number of training segments with optimal costs exceeding 70, representing atypical or inefficient navigation behaviors in training. Interestingly, a higher Removed Curve Count is associated with shorter average times for locating objects during test (estimate = 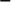 2.7311, *p* = 0.0246). One possible explanation is that taking non-optimal or unconventional routes during training may inadvertently provide participants with a deeper, more comprehensive understanding of the environment. This enhanced familiarity could then translate into more efficient navigation during testing, despite the seemingly inefficient behavior observed in training.

**Table 6:** Summary of scalar regression coefficients for 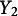 , 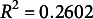

Variables	Covariate	Estimate	Std. Error	*t* value	Pr( 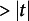 )
Intercept	Intercept	44.5021	10.8432	4.105	6.12e-05
Sex	Female (Baseline)	–	–	–	–
	Male	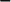 1.2957	3.6840	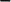 0.352	0.7264
	Other	1.0213	8.2218	0.124	0.9012
Video game experience	Third-person (Baseline)	–	–	–	–
	First-person	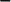 2.5952	4.2853	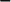 0.606	0.5460
	Neither	0.1854	4.9320	0.038	0.9701
Minecraft skill	Advanced (Baseline)	–	–	–	–
	Intermediate	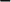 0.1093	5.1296	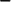 0.021	0.9831
	Novice	8.0958	5.4411	1.487	0.1387
Continuous variables	Gaming hours	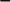 0.3654	0.9132	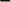 0.400	0.6915
	Removed curves count	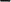 2.7311	1.1964	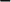 2.282	0.0246
	Age	0.0932	0.4487	0.208	0.8352
Environment	1 (Baseline)	–	–	–	–
	2	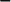 4.6070	4.0217	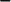 1.146	0.2574
	3	1.2872	4.2761	0.301	0.7645
	4	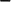 3.7315	4.3602	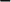 0.856	0.3947
Cluster proportions	Cluster 2	44.8182	49.9001	0.898	0.3708
	Cluster 3	25.8810	47.8012	0.541	0.5891
	Cluster 4	12.3055	17.0134	0.724	0.4728
Time	Time	1.0805	0.2346	4.606	8.18e-06

Notably, average segment time emerged as a significant predictor of 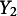 , with a positive association (estimate = 1.0805, *p* = 8.18e-06). This suggests that longer time during training was strongly linked to longer average durations per correct placement. One interpretation is that this variable reflects a participant’s inherent pacing or navigational style. For instance, some individuals may adopt a fast-paced approach in both training and testing phases, while others naturally proceed more slowly and cautiously. These consistent behavioral patterns could explain the strong association between total time spent and average time per successful placement, implying that individual differences in game style—such as speed, thoroughness, or hesitancy—may systematically influence performance efficiency.

**Figure 8:**
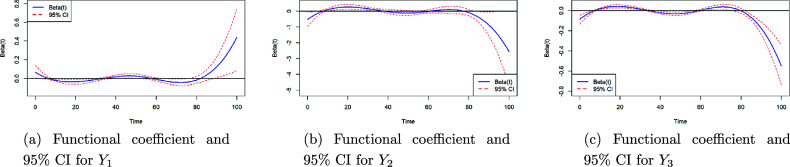
Functional coefficient curves and associated 95% confidence bands for 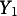 , 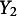 , and 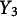 .

However, both the functional covariates of 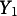 and 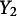 were not significant. Figure 8a and 8b shows the estimated 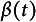 with 95% confidence interval for 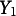 and 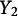 obtained by bootstrap. We can see that most part of the 95% confidence interval for both plots contains zero, which means that they are not statistically significant.

The scalar estimated coefficients for the functional regression analysis on 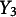 , the average distance from the correct location, are summarized in Table 7. The model explains a moderate proportion of the variance ( 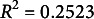 ). A notable effect of Minecraft skill level is observed. Participants in the Intermediate skill group tend to place objects farther from the correct location compared to those in the Expert group (estimate = 0.9158, *p* = 0.0605), while participants in the Novice group perform even worse (estimate = 1.2548, *p* = 0.0199). This finding aligns with expectations, as higher skill levels likely correspond to better navigation and spatial awareness, leading to more accurate placements.

**Table 7:** Summary of scalar regression coefficients for 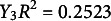

Variables	Covariate	Estimate	Std. Error	*t* value	Pr( 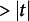 )
Intercept	Intercept	5.1983	1.0546	4.9290	2.0100e-06
Sex	Female (Baseline)	–	–	–	–
	Male	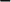 0.3903	0.3449	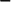 1.1320	0.2594
	Other	0.5923	0.7829	0.7560	0.4505
Video game experience	Third-person (Baseline)	–	–	–	–
	First-person	0.2545	0.4053	0.6280	0.5309
	Neither	0.0097	0.4695	0.0210	0.9836
Minecraft skill	Advanced (Baseline)	–	–	–	–
	Intermediate	0.9158	0.4844	1.8910	0.0605
	Novice	1.2548	0.5335	2.3520	0.0199
Continuous variables	Gaming hours	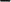 0.0445	0.0871	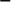 0.5120	0.6096
	Removed curves count	0.0606	0.1148	0.3840	0.7016
	Age	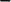 0.0216	0.0427	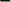 0.5050	0.6142
Environment	1 (Baseline)	–	–	–	–
	2	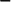 0.6334	0.3776	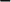 1.6770	0.0954
	3	0.3177	0.4025	0.7890	0.4310
	4	1.1712	0.4172	2.8070	0.0056
Cluster proportions	Cluster 2	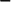 3.1647	1.5982	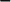 1.9800	0.0493
	Cluster 3	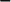 2.7435	4.6854	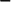 0.5860	0.5590
	Cluster 4	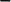 0.9241	4.4559	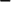 0.2070	0.8359
Time	Time	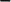 0.0157	0.0230	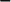 0.6820	0.4964

There is also an environment effect. Compared to Environment 1, which serves as the baseline, participants in Environment 4 place objects farther away (estimate = 1.1712, *p* = 0.0056). This difference across environments is consistent with the findings in the functional clustering analysis (Table 3). Specifically, the proportion of Cluster 1, which represents the best-performing cluster during training, is significantly lower in Environment 4 compared to other environments.

A cluster effect is also observed. A higher proportion of segments in Cluster 2 is associated with more accurate object placement (estimate = 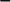 3.1647, *p* = 0.0493). Cluster 2 represents paths that, while less optimal than those in Cluster 1, still maintain relatively straightforward navigation during training. This result can be interpreted through the characteristics of Cluster 2. Paths in this cluster deviate slightly from the optimal trajectory, allowing participants more time to explore the environment while still keeping the target object in mind. This behavior likely helps participants develop a better understanding of the terrain and landmarks surrounding the object’s true location, thereby improving their accuracy during testing.

A notable effect of the functional predictor is observed in the regression on 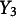 . From the figure of the functional predictor 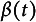 (Figure 8c), we can observe that, although most parts of 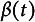 are not statistically significant, the last segment of the functional predictor does exhibit a significant effect. Specifically, the larger the last segment of the average curve, the closer the participant tends to place the objects. This result is somewhat unexpected, as during training, participants who quickly locate and approach the objects are typically considered better performers. However, the behavior represented in the final 20% of the training paths might differ. Participants could move directly and efficiently toward the target object, resulting in a cost difference curve closer to zero. However, they could also take smaller, more tentative steps in the last few blocks before reaching the object. In some cases, they may even deliberately deviate from the optimal route, exploring the area around the object more thoroughly. Such behavior would produce a larger final 20% in the cost difference curve. Participants exhibiting this second type of behavior are likely to develop a better understanding of the specific region surrounding the target object’s true location. This enhanced familiarity with the immediate vicinity of the object could explain their improved accuracy in placing objects closer to their true locations during testing.

In summary, this section extends the analysis of navigation behaviors from segment-level clustering to a participant-level perspective, incorporating functional regression to investigate how training influences test performance. The introduction of the cost difference curve and its functional representation provide a systematic way to capture and quantify navigation strategies, allowing for the identification of key patterns across training and testing phases.

## 4 Discussion

In our study, we demonstrate the application of a novel statistical approach that integrates Dijkstra’s algorithm, cost difference analysis, functional clustering, and functional regression to understand navigation behavior during novel environmental learning and testing using the Minecraft Memory and Navigation Task. Our analysis approach quantified deviations from optimal navigation paths through cost difference by incorporating Minecraft’s unique 3D environmental constraints, such as limited vertical mobility. Functional clustering revealed distinct navigation patterns, ranging from highly efficient strategies to inefficient and wandering behaviors, highlighting the variability in how individuals navigate complex environments. Through functional regression, we demonstrated that training behaviors significantly influence object–location association retention at test, with inefficient navigation patterns during learning associated with poorer spatial accuracy and slower paced navigation during testing. Additionally, individual factors, such as gaming experience and skill level, shaped navigation strategies and outcomes. These findings provide key insights into the relationship between environmental constraints, individual differences, and spatial task performance during navigation in immersive 3D environments.

During training, our findings highlight the significance of navigation efficiency and cluster-based patterns during learning impacting object–location memory. We found four clusters of navigation behaviors during the exploration between object-to-object, ranging from more to less efficient navigation. Participants exhibiting efficient and structured navigation behaviors during training, particularly those represented by Cluster 2, demonstrated improved spatial accuracy in object placement at test. This suggests that moderately efficient but exploratory behaviors during training contribute to a deeper understanding of the environment, enhancing performance in test phases. Given that participants are teleported at test to random locations on the outer edge of the world, this enhanced familiarity acquired during learning likely translates into more efficient navigation during testing, despite the seemingly inefficient behavior observed in training. In contrast, segments associated with inefficient behaviors (Clusters 3 and 4) during training correlated with poorer performance, underlining the importance of optimal navigation strategies.

Key individual factors, such as Minecraft skill level, sex, and prior gaming experience, also played a role. In line with prior studies demonstrating an association between hours spent playing video games and improved spatial skills, we found that higher skill levels consistently aligned with better performance metrics, including more accurate and efficient navigation (Murias et al., 2016; Yavuz et al., 2024). Surprisingly, those who play first-person games performed worst is surprising given that Minecraft is predominantly a first-person game. This could possibly be due to the broader spatial awareness developed through third-person perspectives. Third-person perspective can provide a more comprehensive and broader perspective while learning an environment (Burigat et al., 2017), thus it is possible that players with third-person game experience develop a better understanding of the spatial environment, thereby enhancing their retention of the object location associations. One prior study (Ventura et al., 2013) found participants with more first-person game experience performed better; however, they did query about third-person experience. These results emphasize the interaction between individual factors, prior experience, and task-specific demands.

A novel insight emerged regarding the impact of inefficient training behaviors at testing. Participants with a higher count of removed curves (segments with suboptimal costs) displayed shorter average times to navigate to an object’s location at testing, suggesting that exploratory or unconventional routes during training may foster a more comprehensive understanding of the environment. Additionally, the final 20% of the functional predictor in 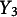 ’s regression revealed that participants who exhibited tentative or exploratory navigation behaviors near target objects during training were more likely to place objects closer to their correct locations in testing, highlighting the value of local exploration. Our finding is in line with research demonstrating that across the lifespan, greater exploration, more efficient, and active navigation enhances memory for spatial memory accuracy (Farran et al., 2022).

Future research directions should focus on several key areas. A more detailed analysis of test behaviors using methods similar to those applied during training could yield valuable insights. Specifically, examining turning patterns and decision-making processes during the test phase, akin to how they were analyzed in training, could help identify consistent behavioral strategies. Additionally, it is important to address participants who may not take the test seriously and place objects at random locations rather than based on memory, as these behaviors could skew results and reduce the robustness of findings.

In addition, future extensions of our methodology could incorporate recent advances in robust or local functional clustering. For example, methods, such as SaS-Funclust (Centofanti et al., 2021) enable simultaneous clustering and variable selection through sparse, smooth mixture modeling, while techniques like funLOCI (Di Iorio & Vantini, 2024) identify clusters based on localized functional features rather than global patterns. These approaches may help uncover navigation subtypes that are temporally or spatially restricted, i.e., patterns that may be masked when averaging cost-difference curves across training segments. Exploring such localized or hierarchical clustering strategies could enhance sensitivity to subtle variations in navigation behavior and provide finer-grained predictors for memory outcomes.

Environment-specific features, such as landmarks and terrain effects, were not sufficiently considered in this study. Unique landmarks like trees, fences, and rivers, as well as distinct terrain types, such as deserts, mountains, and grasslands, likely enhance participants’ memory of specific locations, thereby improving performance. Future studies could explore how these environmental cues interact with navigation strategies and memory recall. Furthermore, the angle at which participants approach objects was overlooked. Certain approach angles, influenced by terrain features or starting locations, may significantly impact object placement accuracy and navigation efficiency.

Statistical challenges emerge when evaluating movement behavior in 3D environments, in which the spatiotemporal information is highly auto-correlated. Furthermore, constraints within the video game, such as how high one can jump or fall, impact the weighting of movement decisions. Applying Dijkstra’s algorithm to navigation is particularly suited for navigation and network analysis as the algorithm models the complexities of spatial constraints. Our application of this algorithm to understand movement in Minecraft builds on graph-based models to analyze trajectory data (Chrastil & Warren, 2014; Spaccapietra et al., 2008). In line with graph theory, movement is modeled using graphs, where locations are depicted as nodes and movements as edges. More advanced and flexible modeling methods, such as graph-based representation learning (Elayam et al., 2022; Martin et al., 2023) and deep learning, could further enhance the analysis of navigation behaviors in 3D environments. These models have the potential to uncover deeper insights into spatial decision-making by adapting to multi-session data and capturing longitudinal behavioral changes more effectively.

One limitation of this study was the immediate nature of our data. We utilized data from tests conducted immediately after learning, which limits the scope of our analyses to short-term memory effects. The original MMN task evaluated performance over a 12-hour break of sleep or wake, finding similar average navigation behaviors over both intervals (Simon et al., 2022). Future research should further identify subtle differences in navigation strategies, compared to the broader, generalized patterns observed in the original study, and explore how these differences evolve over time. Additionally, the sample size and diversity in video game experience could be improved. While 182 participants provide a substantial dataset, broader and more representative sampling across different age groups from children through older adults and other gaming experiences could improve the generalizability of our findings. Determining additional individual factors, such as engagement and motivation during the experiment, would also address potential sources of bias in the results. Future research can also expand to mental health or medical disease populations to determine the impact of these symptoms on spatial navigation and memory.

Functional data clustering has been extensively studied, with early foundational work adopting functional principal component (fPC) decomposition within model-based frameworks (James & Sugar, 2003), or combining spline-based representations with centroid-type clustering algorithms (Abraham & Cornillon, 2003; Giacofci et al., 2013). These approaches often assume that each function arises from a Gaussian process or follows a specific low-dimensional representation. More recently, robust extensions, such as the trimmed *K*-means (García-Escudero & Gordaliza, 2005), DBSCAN and HDBSCAN (Campello et al., 2013), and sparsity-enforcing models like SaS-Funclust (Centofanti et al., 2021) have been introduced to mitigate the effects of outliers and high variability in complex datasets.

Our method fits within the centroid-based spline clustering paradigm, using cubic B-spline basis coefficients and *K*-means clustering (Jacques & Preda, 2014). While simple and effective, this approach may be limited in capturing local or multi-scale heterogeneity. Future work could build on robust or hybrid approaches—e.g., combining spline representations with mixture models or integrating region-specific behavior detection as in funLOCI (Di Iorio & Vantini, 2024). These alternatives offer promising directions for improving sensitivity to behavioral subtypes embedded in navigation trajectories.

## Figures and Tables

**Figure A1:**
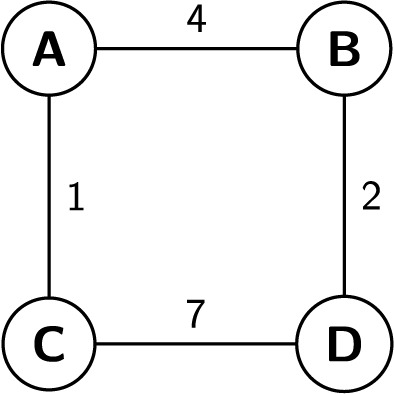
Weighted graph from source A.

**Figure C1:**
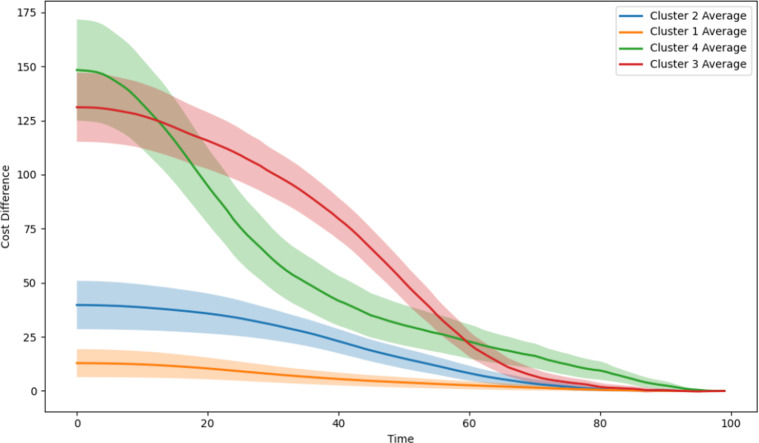
Cluster-averaged trajectories and associated confidence bands using FPCA-based clustering.

**Figure D1:**
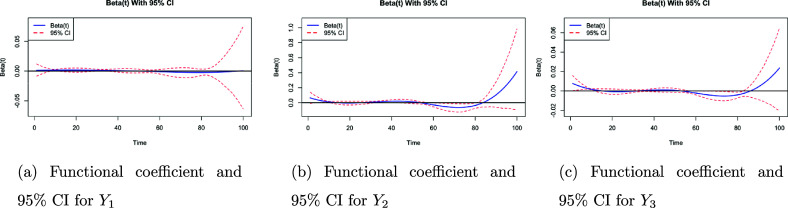
Estimated functional coefficient curves for the merged cluster 3 & 4 average cost difference curve as covariate, along with 95% bootstrap confidence bands, for the three test outcomes 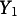 , 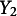 , and 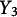 .

**Figure E1:**
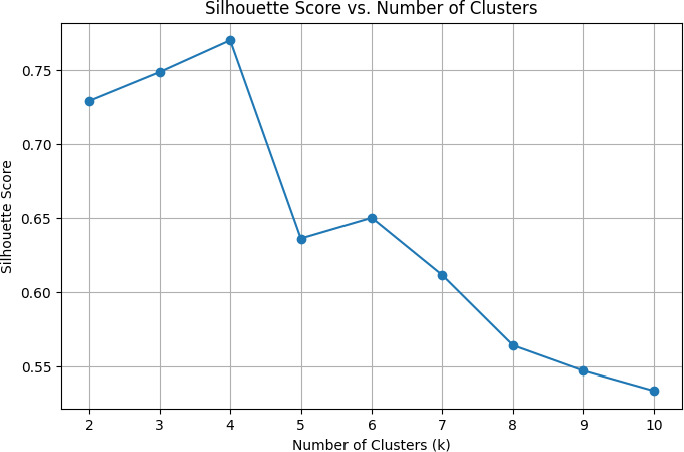
Silhouette score plot for clustering results in Section 3.4.

## Appendix A: Dijkstra’s Algorithm

Dijkstra’s algorithm guarantees the optimal path from a source node to all other nodes, assuming non-negative edge weights. It proceeds by maintaining a priority queue of nodes ordered by tentative distance from the source, updating distances as shorter paths are discovered. This makes it well suited for environments such as Minecraft, where elevation, obstacles, and terrain difficulty significantly affect traversal cost. The algorithm proceeds iteratively: at each step, it selects the unvisited node with the smallest tentative distance, updates its neighbors, and marks it as visited. This process continues until all nodes are finalized or the destination is reached. The following example demonstrates this step-by-step (Figure A1).

**Table A1:** Tentative distances at each step

Step	Visited	dist(B)	dist(C)	dist(D)
Init	none			
1	A	4	1	
2	C	4	1	**8** (=1+7)
3	B	4	1	**6** (=4+2)
4	D	4	1	6

**Explanation**: In the example above, the algorithm begins at node A (start point), where the tentative distances to B and C are initially set to 4 and 1, respectively. Node C is visited next because it has the lowest tentative distance. From C, the path to D is computed as 1+7=8. However, when node B is subsequently visited, a shorter path to D is found (4+2=6), and the tentative distance is updated accordingly. Once we finish the Table 8 construction (after Step 4), we obtain the shortest distance, hence the path from node A to each of the nodes B, C, and D. For example, we know the shortest distance from A to D is 6 based on the number 6 at the lower right corner of the table and this path is only feasible after we visit node B; hence, the path is A-B-D.

## Appendix B: Sensitivity analysis on clustering

To assess the sensitivity of clustering results to the number of spline basis functions used in Eq. (2), we varied the number of basis functions *K* and evaluated clustering consistency using the adjusted Rand index (ARI), which takes values between 0 and 1 with bigger values indicating a higher degree of consistency. We used  as the baseline and compared it with alternative values.

The results in Table B1 suggest that the clustering results are generally robust to moderate changes in the number of basis functions, especially when  , where the ARI is always above  . These results confirm that our clustering results are not sensitive to the choice of *K*.

**Table B1:** Adjusted Rand index (ARI) between cluster results obtained with different values of *K* and the baseline

Alternative *K*	4	6	8	12	15	18	20
ARI vs.	0.7088	0.8682	0.8678	0.9226	0.9028	0.8993	0.8847

## Appendix C: Clustering using FPCA approach

Besides B-spline expansion, functional principal component analysis (FPCA) is another popular approach in functional data analysis, especially for capturing transitions and curvature in the data. Here, we rerun the clustering analysis in Section 3.4 using FPCA. We choose the first three principal components as they explain over 95% of the variance in the data. Each participant’s cost difference curve was then projected onto these components, yielding a low-dimensional representation (i.e., FPCA scores) that captures the dominant modes of variation. We then performed *K*-means clustering on the FPCA scores. The resulting clusters, summarized in Figure C1, indicate that the average trajectories for four clusters were very similar to those obtained using B-spline expansion in Figure 4a. In other words, our interpretations of navigation behavior do not depend on the choice of functional basis for the cost difference curves. These results suggest that FPCA offers a valuable alternative to spline basis clustering, with comparable interpretability and performance.

## Appendix D: Functional regression analysis based on cluster-specific functional covariates

In Model (3), we only consider one functional covariate, that is, the average cost difference curve from low-cost segments. It is possible to explore other choices, e.g., using average cost difference curves from each of the clusters. We conduct this exploration in this section.

Since Cluster 3 and 4 segments only represent less than 5% of the total segments in training, we combine them together and use the average cost-difference curve. This merging step also helps us keep a reasonable sample size (among the 182 participants, only 36 of them have both cluster 3 and cluster 4 curves). We also consider the average curves from cluster 1 or 2 when available for each participant. The regression model is fitted using these new functional covariates and the same set of scalar covariates as before.

The estimated functional coefficient curves for the merged cluster 3 and 4 curves are shown in Figure D1 for each of the three outcomes, together with their  confidence bands obtained by bootstrap. We cannot find any statistically significant association between the test outcomes and the cluster 3  4 averaged curves. As a result, we decide not to include them in our analysis. For cluster 1 and 2 curves, we have observed a strong collinearity between them. Based on these three reasons, we choose to include only the average cost difference curve for the low-cost segments, that is, we simply ignore the cluster 3 & 4 curves because of lack of data and high noise; and we choose to combine cluster 1 & 2 curves since they share very similar trend information.

## Appendix E: additional results

In Figure E1, the elbow plot of Silhouette scores suggests that a cluster number of  is a reasonable choice for the clustering results in Section 3.4.
